# Cancer Research in Vulnerable Populations: A Call for Collaboration and Sustainability From MENAT Countries

**DOI:** 10.1200/GO.23.00201

**Published:** 2023-12-14

**Authors:** Marwan Tolba, Mac Skelton, Zahi Abdul Sater, Ibtihal Fadhil, Ali Al-Zahrani, Tezer Kutluk, Kamal Akbarov, Ali Taher, Richard Sullivan, Layth Mula-Hussain

**Affiliations:** ^1^ Department of Radiation Oncology, Dalhousie University, and Cape Breton Cancer Center, Sydney, Nova Scotia, Canada; ^2^ Institute of Regional and International Studies, American University of Iraq, Sulaimani, Iraq; ^3^ Global Oncology Group, King's College London, London, United Kingdom; ^4^ College of Public Health, Phoenicia University, Mazraat El Daoudiyeh, Lebanon; ^5^ Global Health Institute, American University of Beirut, Beirut, Lebanon; ^6^ Eastern Mediterranean NCD Alliance, Dubai, United Arab Emirates; ^7^ King Faisal Specialist Hospital and Research Centre, Riyadh, Saudi Arabia; ^8^ Gulf Center for Cancer Control & Prevention, Riyadh, Saudi Arabia; ^9^ Department of Pediatrics, Division of Pediatric Oncology, Hacettepe University Faculty of Medicine & Cancer Institute, Ankara, Turkey; ^10^ Division of Human Health, International Atomic Energy Agency, Vienna, Austria; ^11^ Hematology & Oncology, Naef K. Basile Cancer Institute, American University of Beirut Medical Center, Beirut, Lebanon; ^12^ King's College London & Guy's Comprehensive Cancer Centre, Global Oncology Group & Institute of Cancer Policy, Centre for Conflict & Health Research, London, United Kingdom; ^13^ College of Medicine—Ninevah University, Mosul, Iraq

## Abstract

**PURPOSE:**

Cancer is a major burden across Middle East, North Africa, Türkiye (MENAT). Many MENAT countries experience multiple conflicts that compound vulnerabilities, but little research investigates the linkages between vulnerability and cancer research. This study examines the current level and the potential for cancer research among vulnerable populations in the MENAT region, aiming to provide direction toward developing a research agenda on the region's vulnerable populations.

**METHODS:**

Expert-driven meetings were arranged among the 10 authors. After obtaining institutional review board approval, a self-administered online survey questionnaire was circulated to more than 500 cancer practitioners working in 22 MENAT countries.

**RESULTS:**

Two hundred sixteen cancer practitioners across the MENAT region responded. Fifty percent of the respondents identified clinical research in vulnerable patients with cancer as a significant issue; 21.8% reported previous research experience that included vulnerable populations, and 60% reported encountering vulnerable populations in their daily clinical practice. The main barriers to conducting research were lack of funding (60%), protected time (42%), and research training (35%). More than half of the respondents believed that wars/conflicts constituted an important source of vulnerability. The most vulnerable cancer populations were the elderly, palliative/terminally ill, those with concomitant mental health-related issues, those with other chronic illnesses, and socioeconomically deprived patients.

**CONCLUSION:**

Results support that a major effort is needed to improve cancer research among vulnerable cancer populations in the MENAT region. We call for interdisciplinary research that accounts for the region's unique, compounding, and cumulative forms of vulnerability. This cancer research agenda on different vulnerable populations must balance sociobehavioral studies that explore sociopolitical barriers to quality care and clinical studies that gauge and refine treatment protocols. Building a research agenda through collaboration and solidarity with international partners is prime time.

## Teaser

Efforts are needed to improve cancer research among vulnerable cancer populations in MENAT countries

## INTRODUCTION

Cancer is a major global health challenge, with disparities in incidence, morbidity, and mortality between different geographical regions, populations, and income levels. Low- and middle-income countries (LMICs) will bear the brunt of the increasing cancer burden.^1-6^ Unfortunately, the disparate cancer burden is not being addressed by locally driven cancer research in LMICs, which is further compounded by the limited transferability of knowledge gained in high-income countries. The Middle East, North Africa, Türkiye, *including Cyprus*, (MENAT) region is no exception to the global disparities in cancer. The region, which consists of 25 countries, most of which are considered LMICs, is heterogeneous in its socioeconomic, political, and cultural dimensions, resulting in varying cancer incidence and mortality rates (Appendix 1 and Appendix Table A1).^7,8^ Many countries in the region, including Iraq, Syria, Yemen, Sudan, Lebanon, and Occupied Palestinian Territory, are affected by recurrent and endemic conflicts, which led to weakened and fragile health systems, further increasing vulnerability in resident populations.^2,4,5,9,10^ Consequently, cancer care and outcomes have been severely affected for minorities, ethnic groups, refugees, and internally displaced populations. Yet, our understanding of cancer research in these vulnerable populations remains limited, with noticeable gaps in sociobehavioral studies examining care barriers and clinical research assessing variable treatment responses.^4,11-17^

**CONTEXT**

**Key Objective**
This study is the first to investigate the prospect of advancing a cancer research agenda focusing on vulnerable populations in the Middle East, North Africa, Türkiye (MENAT) region. The primary goal of this study was to explore how MENAT practitioners understand the distribution and characteristics of the region's vulnerable populations, in addition to delineating barriers to conducting research including such populations.
**Knowledge Generated**
Research on vulnerable populations in the MENAT region remains limited because of lack of funding, protected time, and training in research methods. Conflict forms a crucial part of vulnerability in the MENAT region, leading to a compounded impact on already vulnerable groups.
**Relevance**
We call for a robust interdisciplinary research agenda that accounts for the unique and cumulative forms of vulnerability in the MENAT region. This research agenda must balance sociobehavioral studies that explore barriers to quality care and clinical studies that gauge and refine treatment protocols.

What would a robust cancer research agenda focused on the vulnerable populations of the MENAT region entail? Recent research on vulnerable populations in other regions has started the research by isolating one specific vulnerable population for examination,^18-20^ but this approach is only possible in a context in which the most pressing forms of vulnerability are already established (Appendix 2). Because the notions of vulnerability and vulnerable population are relatively new to cancer practitioners and researchers in the MENAT region, the primary goal of this study was to explore how MENAT practitioners understand the region's landscape of vulnerability and how they might align or diverge from the categories of vulnerable populations as delineated in other research.^21^ By examining the possible interplay between MENAT's protracted conflicts and vulnerability and evaluating local research capacity and resources, we aim to identify areas for improvement and highlight context-specific challenges. Our goal is to outline a research agenda focusing on vulnerable populations, creating a roadmap for sustainable and collaborative cancer research in the MENAT region.

## METHODS

### Survey Development

Recurrent expert meetings among the coauthors were held to codevelop the study's objectives. In these meetings, the group identified the challenges and barriers that negatively affect cancer research capacity and clinical care outcomes in the MENAT region, particularly among vulnerable populations, including those affected by conflict. The authors modified and expanded on lists of vulnerable populations included in other research,^21^ with the aim of capturing region-specific vulnerabilities in MENAT that may differ from other regions. Informed by the expert-driven meeting, a 40-item online survey questionnaire was codeveloped. A pilot survey was sent to a limited group of 15 respondents to validate the survey questions, and a final questionnaire version was run using LimeSurvey Cloud version 5.3.25 (Hamburg, Germany). The survey comprised 41 questions covering five domains: demographic and general information, cancer care in clinical practice, research capacity and research among vulnerable populations, perception of vulnerability, and infrastructure and resources for cancer management. The live run time of the survey was 15 days in 2022, from July 17 to July 31 inclusive, and two reminder notes were sent to candidates on different occasions before the closure of the survey.

### Sampling

Purposive and snowball sampling methods were chosen and adopted for optimal information gathering in such a challenging environment to reach a difficult-to-reach target cohort. This technique was used to obtain reliable data from representatives who were knowledgeable about different clinical settings and communities across the MENAT. Nonrandom methods were preferred because of low and unreliable responses from previous experiences. Purposive and snowball sampling methods are appropriate for investigating sensitive and stigmatized topics in communities with marginalized and vulnerable populations^22-25^

The survey was circulated among various specialists/stakeholders involved in clinical cancer research and care working in several MENAT countries. The participants were selected from the authors' network, which included medical, clinical, radiation, and surgical oncologists, besides nutritionists, nurses, palliative care specialists, etc. In the invitation email, participants were encouraged to circulate the survey to their network to increase participation. Ultimately, the survey was disseminated to more than 500 cancer care practitioners in the MENAT region.

### Inclusion/Exclusion Criteria

Responses from participants who did not specify a country of practice, selected a country of practice outside the MENAT region, or provided a specialty that is not related to oncology were excluded.

### Institutional Review Board Approval and Consent

The study proposal was submitted to and has been approved by the Institutional Review Board at the American University of Iraq, Sulaimani. In addition, a welcome message, including the ethical and logistical details and a consent form, was added to the survey.

## RESULTS

### Respondents Profile

The survey, which was sent to more than 500 cancer practitioners, received a total of 304 responses. However, after excluding 88 responses that did not meet the inclusion criteria, the final number of oncologists working in the MENAT region who completed the survey was 216 (71.1% of the total respondents). Of the 216 respondents, 61.6% were male and 47% were in the age group of 35-44 years. The most common oncology specialties among respondents were radiation oncologists (35%), medical oncologists (32%), and then clinical oncologists (13%), oncologists who are trained to deliver radiotherapy and chemotherapy and are practicing oncology with this scope. Respondents reported working in 22 countries and territories across the MENAT region with Iraq, Türkiye, Egypt, Saudi Arabia, and Algeria constituting 26.4%, 11.6%, 8.8%, 8.3%, and 5.6% of the total group, respectively (Table 1). Most respondents reported working at public institutions and/or university hospitals while about 25% reported working at private, military, nongovernmental organizations, or other care facilities. In addition, 28% of the practitioners responded that they have 11-20 years of experience and 27% with 5 years of practice or less.

**TABLE 1:**
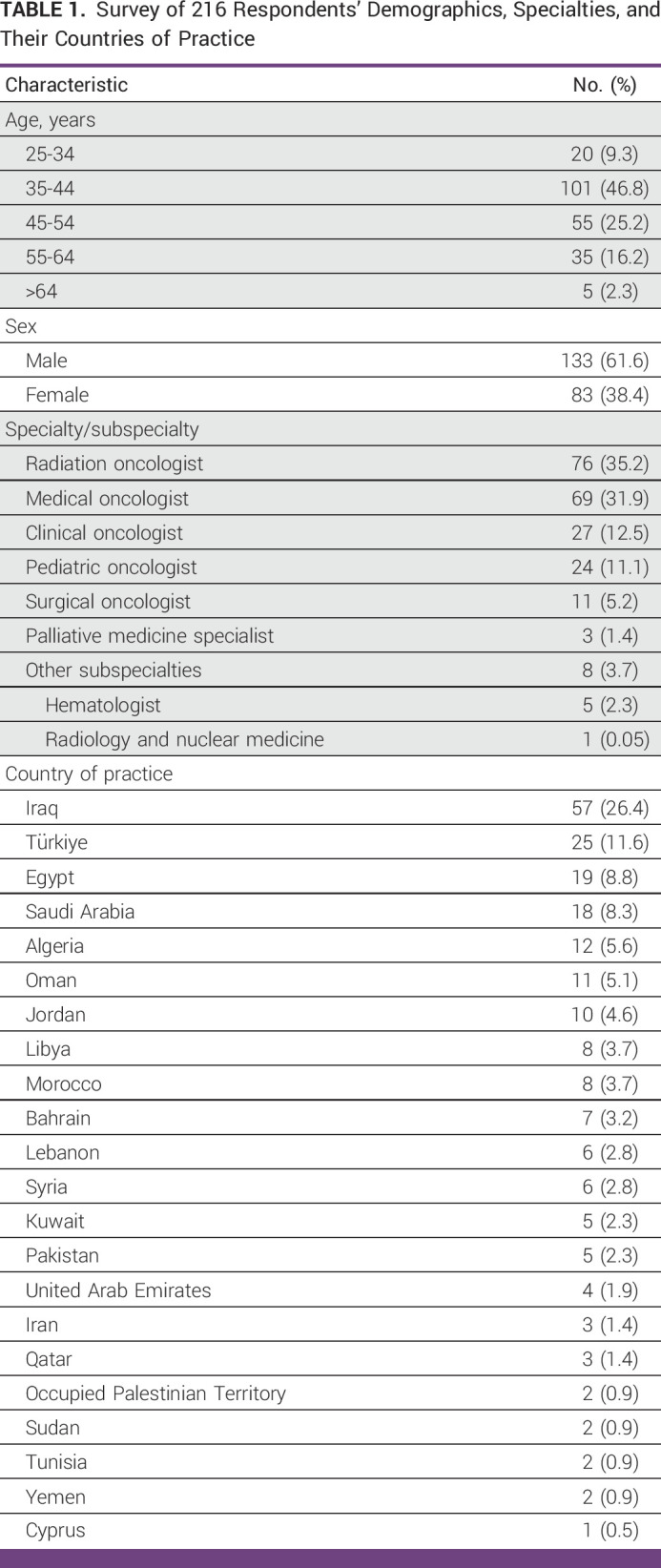
Survey of 216 Respondents' Demographics, Specialties, and Their Countries of Practice

### Status and Challenges of Cancer Research Among Vulnerable Populations

Of the 216 respondents, 67% reported previous research experience (Table 2). However, the majority of experience was in basic and noninterventional cancer research, with only 21% having experience in interventional cancer clinical trials. Furthermore, more than half of respondents were unaware of any cancer-related clinical studies being conducted in their workplaces. Although more than half of the respondents considered research to be crucial to their workplace practice, 20.8% reported that research is not mandatory. Prestige and promotion were the main incentives for research, reported by 61% and 59%, respectively, with only 14% reporting salary incentives. Key challenges for conducting research included lack of funding (60%), protected time (42%), and research training (35%). Only 23% of respondents reported that their facility incentivizes research, highlighting the need for increased support and resources for cancer research in the MENAT region.

**TABLE 2:**
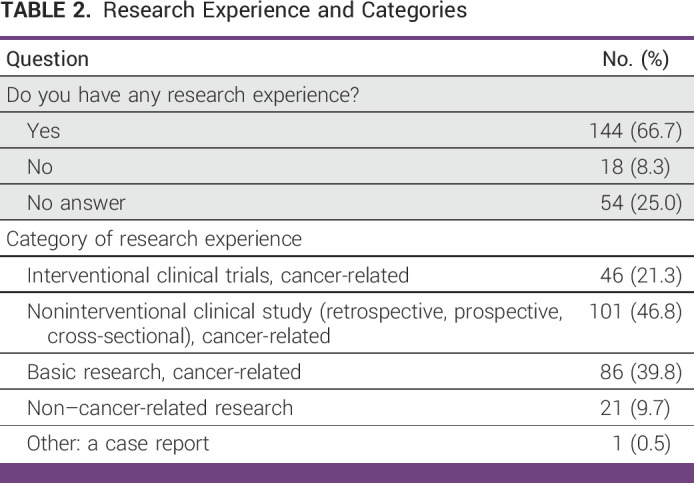
Research Experience and Categories

The qualitative data highlighted two themes related to the challenge of cancer research among vulnerable populations: First, the data indicated widespread frustration because of a perceived lack of support for research. In a comment that reflected many others, one oncologist noted: “The facility doesn't provide funds to support research. [The] researcher must pay from their own pocket to support their research efforts.” Other oncologists noted a lack of incentives, with one specifying that “no protected time is offered, and afterward no recognition.” The lack of funding and institutional support also means that there is a lack of well-trained staff to guide research projects. One respondent elaborated on this point: “The supervisors for research projects do not have the basic understanding of research process and how to support and educate new trainees.” These findings are consistent with existing literature,^26^ which emphasized the disruptive effects of conflict on cancer provision and research in the region, including unsafe environments, fragmented facilities, and the migration of health care professionals.

Second, oncologists in this study repeatedly stressed that particular social and cultural dynamics often impede both sociobehavioral studies and clinical trials. In a cultural context in which withholding information about cancer is commonplace, obtaining consent for research would be extremely complex, they emphasized. One respondent noted: “The problem is sometimes [the] patients' relatives try to hide the full details of the clinical situation [from] the vulnerable patient.” Other respondents indicated that many patients simply would not see the value of participation in research because of cultural reasons. Although cultural norms may play a role in discouraging participation in research, it is likely that this lack of willingness is also a function of the widespread distrust of doctors in conflict-affected areas of the MENAT. Studies have shown how wars in Iraq, Syria, and other parts of the Middle East region have resulted in a gradual deterioration of trust between patients, doctors, and their families^10,27,28^—a fact that necessarily would complicate conducting sensitive research among vulnerable patients.

### Perception of Vulnerability

**FIG 1:**
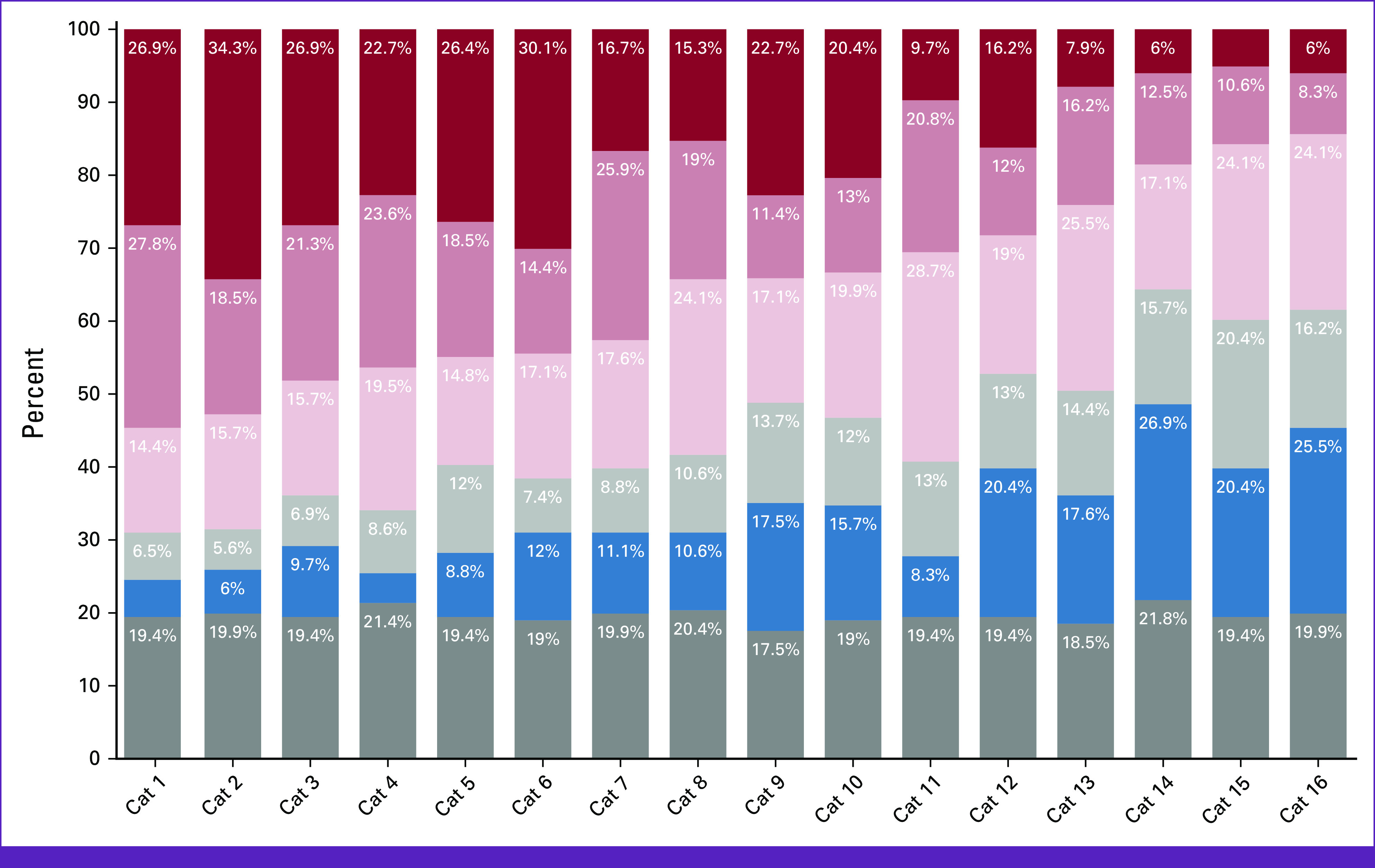
Perceived ranking of vulnerability by cancer practitioners. Cat, categories.

Approximately 60% of cancer practitioners participating in the survey reported seeing vulnerable populations (Appendix Table A3) during their daily practice. Only 22% reported conducting research with vulnerable populations (Appendix Table A2). When asked to assess the relative vulnerability of 16 different vulnerable populations according to relative severity (Table 3), the most vulnerable groups (with scores of 4-5) were seniors and geriatric patients, terminally ill patients, patients with mental health-related comorbidities, socioeconomically deprived or socially isolated patients, chronically ill patients, and patients with limited health literacy or physical disability, corresponding to 54.7%, 52.8%, 48.2%, 47.2%, 44.9%, and 42.6% of the responses (Fig 1 and Table 3). The lowest ranked categories, with scores of 1-2, were people of the LGBTQ+ community, veterans, divorced individuals or widowed women, prisoners, ethnic minorities, and pregnant patients, corresponding to 42.6%, 41.7%, 40.8%, 33.4%, 32%, and 30.5% of the responses, respectively. Nine percent of participants suggested categories of vulnerable populations that should be added to the existing list, whereas 13.4% chose to remove some categories from the list we provided (Appendix Table A3).

**TABLE 3:**
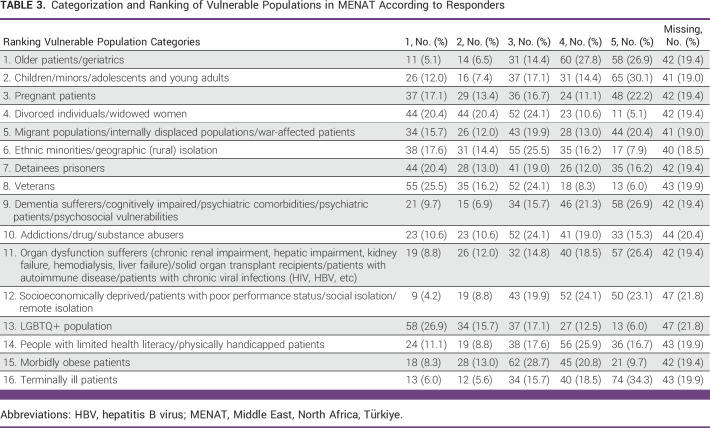
Categorization and Ranking of Vulnerable Populations in MENAT According to Responders

The qualitative results shed light on these rankings and suggested omissions. For the issue of conflict and vulnerability, roughly half of the respondents (n = 109) agreed that recent wars/conflicts worsened the conditions for vulnerable populations in accessing and receiving cancer treatments. The top war-related conditions complicating cancer care were loss of lives and/or jobs, sale of homes for treatment cost, internal displacement or migration, war-related injuries, and war-related psychological trauma, corresponding to 49.1%, 38.9%, 38.4%, 37.8%, and 37.5%, respectively. The qualitative data confirmed that conflict was seen as an important source of vulnerability in the MENAT region—the one that exacerbated other forms of vulnerability. One respondent noted previous research experience with pediatric cancer groups escaping from war and another had experience researching the conflict-affected terminally ill. Indeed, vulnerable populations on the basis of age or comorbidity may also be displaced or suffering war-related distress, a finding that highlights the need for interdisciplinary research teams and mixed methods that are sensitive to these overlapping forms of vulnerability. Although a subset of participants had no ongoing conflict in the country in which they practiced, they either hosted affected populations or traveled between countries for cancer care.^9,10^ The results indicate that specific vulnerable groups in the MENAT region may be subject to cultural biases that obscure clinicians' perceptions of vulnerability (Appendix 3). A small but notable number (21) of respondents expressed that LGBTQ individuals do not qualify as vulnerable patients with cancer, with some explicitly writing in the qualitative responses that LGBTQ must be removed from the categories of vulnerability. Such responses may reflect societal attitudes and prejudices toward LGBTQ individuals in the MENAT region. Indeed, existing research on the subject suggests that religious, cultural, and legal aspects may pose barriers to equitable health care access for LGBTQ populations in the region.^29^

### Priorities for Future Research on Vulnerable Populations

Qualitative results indicate that improving the access of vulnerable populations to cancer services must be a top research priority. When asked to explain why cancer research among vulnerable populations is important, respondents emphasized the need to improve access to care. In a comment that reflected many others, one respondent noted: “Poor refugees cannot afford expensive treatments, [and] hundreds of my patients died due to lacking money for treatment.” Although researchers in the MENAT region have begun exploring the problem of catastrophic expenditure for war-affected patients with cancer,^9^ the research on this subject requires further cross-regional elaboration. Other respondents highlighted the need for research to aid in improving clinical management, noting that vulnerable patients are usually kept out of prospective trials, which actually limits the validity of the results.

## DISCUSSION

The results highlight the multilayered challenges to conducting cancer research among vulnerable populations in the MENAT region.

Economic, social, and even geopolitical challenges pose barriers to research and cancer management in different MENAT countries.^30-33^ Compared with the global community, Arab and MENAT countries contribute very little cancer research in comparison.^12,34^ In the MENAT region, the average scientific output published locally over the past 5 years was 4.6 publications per practitioner, compared with 7.1 published internationally. Respondents in this study reported an average of 3.0 active research projects in progress per practitioner. The mean publications of respondents from different countries varied widely; however, these results were likely influenced by several country-specific factors and should also be interpreted with caution (Appendix Table A4). Previous reports have suggested that conflict, political disputes, and low research quality are the main reasons for this gap.^35-37^ Others have attributed this gap to different funding issues and neglected investment in research infrastructure.^10,15^ Availability of data and population-based cancer registries are considered major pillars to reliably assess the burden of cancer in a given region.^38^ In spite of increasing cancer burden among people in LMICs, particularly in the MENAT region, cancer registration in this region is not well-established and is hindered by a variety of difficulties.^4,5^ Efforts to improve cancer registration and control in the MENAT region should be prioritized.

Most respondents in this study reported experience with research and considered research to be essential to workplace practice. This highlights the main challenges faced by cancer practitioners in the region, namely lack of funding, protected time, and training in research methods. The lack of research among vulnerable communities is not due to a lack of clinical contact. In our survey study, most respondents reported witnessing vulnerable populations during their daily practice; however, only a quarter of cancer practitioners in the MENAT region worked on research that actively included vulnerable populations (Table 4).

**TABLE 4:**
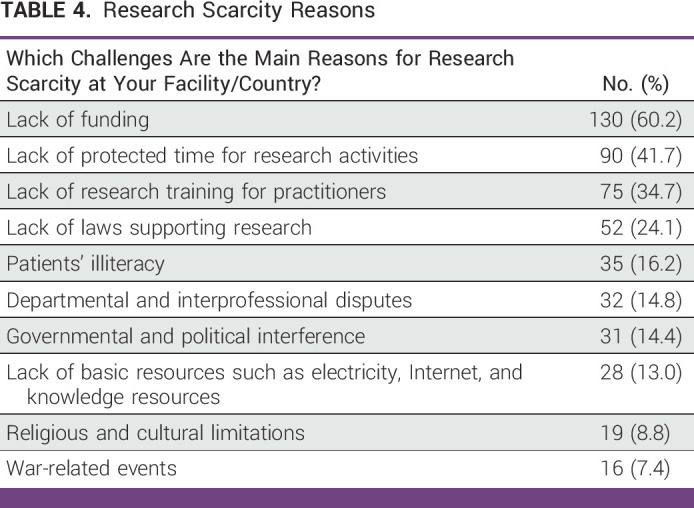
Research Scarcity Reasons

The largest percentage of respondents hailed from Iraq, Türkiye, Egypt, Saudi Arabia, and Algeria. These countries constitute 50% of the MENAT population (272,224,148 of 522,195,083) and hence have a considerable number of practitioners. The five countries represent a balance between conflict-affected and relatively stable MENAT contexts. Practitioners from countries such as Iraq, who have experienced protracted war and conflict, are especially important informants as they have firsthand experience of the vulnerabilities and crises that affect their practice and daily lives. Additionally, according to the United Nations refugee agency, Türkiye currently hosts the largest number of refugees globally, with more than 3.6 million registered Syrian refugees and nearly 320,000 persons of concern from other nationalities. In contrast, the oil-rich Saudi Arabia has engaged in military operations elsewhere in the region but remained economically and politically stable at home.

This study has several strengths; to our knowledge, this study is the first to examine the status and potential for cancer research among vulnerable populations in the MENAT region. However, the study is limited by the inability to cover all subspecialties in the region because of logistical and geopolitical challenges and by the limitations of a survey-based approach. The authors recognize the potential bias risk associated with the utilization of the purposive and snowball sampling methods. This arises from the unevenness of selection among members of the target population and the potential influence of preexisting relationships or shared characteristics among participants. However, proactive and thorough measures were undertaken to address the bias risk. This was achieved through the precise delineation of our research objectives and goals. Additionally, a comprehensive set of criteria was considered during the participant selection process. This facilitated the enlistment of a diverse cohort, encompassing a broad spectrum of cultures, backgrounds, beliefs, perspectives, and experiences.

Because the notion of vulnerable populations^39^ is a relatively new topic among cancer practitioners in the MENAT region, there was considerable debate among the authors as to the appropriate delineation of the 16 vulnerability categories and subcategories. Although it is likely that another group of coauthors would have likely derived a different list, the aim of the study has not been to establish definitive or precise markers of vulnerability. Rather, the objective has been to provide the initial direction for a research agenda on the basis of the perceptions of oncologists. The results indicated that there is a critical need for increased collaboration and cooperation between researchers, practitioners, and policymakers in the MENAT region and the international community to enhance cancer research among vulnerable populations in the region.

From the findings of the survey, we propose a robust cancer research agenda for the vulnerable populations of the MENAT region that integrates multiple insights from our results. First, a priority should be addressing institutional barriers that cancer practitioners encounter in conducting research, such as limited funding, lack of protected time, and insufficient research training. Second, the high vulnerability perceived in the elderly and terminally ill patients and those with mental health-related comorbidities, low socioeconomic status or social isolation, chronic illness, limited health literacy, or physical disability emphasizes the multifaceted nature of vulnerability. These categories can be perceived as most vulnerable because of the complex intertwining of socioeconomic, physical, and mental health challenges that these populations face. These factors often contribute to reduced access to care, poor understanding of health information, and thus, less optimal treatment outcomes.

Third, it is evident that conflict forms a crucial part of vulnerability in the MENAT region, leading to a compounded impact on already vulnerable groups. Therefore, a comprehensive research agenda should examine the intersectionality of vulnerabilities within the war-affected populations to provide better cancer care to these groups. Furthermore, our findings may also highlight certain cultural biases present in the region that influence perceptions of vulnerability and affect the health care access of certain groups. These biases must be considered when formulating a research agenda, and efforts should be made to address these gaps to ensure that no group is left behind. Additionally, the research agenda should place equal emphasis on sociobehavioral studies that explore sociopolitical barriers to care and clinical studies that refine treatment protocols for different vulnerable populations, acknowledging that both facets are critical for enhancing cancer care in the region. Finally, the low participation rate of patients and families in research in the MENAT region presents a unique challenge, indicating a need to foster trust and promote the value of research participation among these populations.

In conclusion, this study highlights the difficulties and challenges faced by cancer practitioners in the MENAT region in conducting research among vulnerable populations. Lack of research funding and training of cancer practitioners in the MENAT region are major factors negatively affecting cancer research. Addressing cancer disparities in the MENAT region is a complex and pressing issue that requires the collective efforts of researchers, practitioners, policymakers, and the international community. We call for a robust interdisciplinary research agenda that accounts for the unique and cumulative forms of vulnerability in the MENAT region, which for many patients with cancer are compounded by protracted wars and displacements. This research agenda on different vulnerable populations must balance sociobehavioral studies that explore sociopolitical barriers to quality care and clinical studies that gauge and refine treatment protocols. By working together and providing the necessary resources, we can improve cancer research outcomes for the most vulnerable populations in the region.

## Figures and Tables

**TABLE A1:**
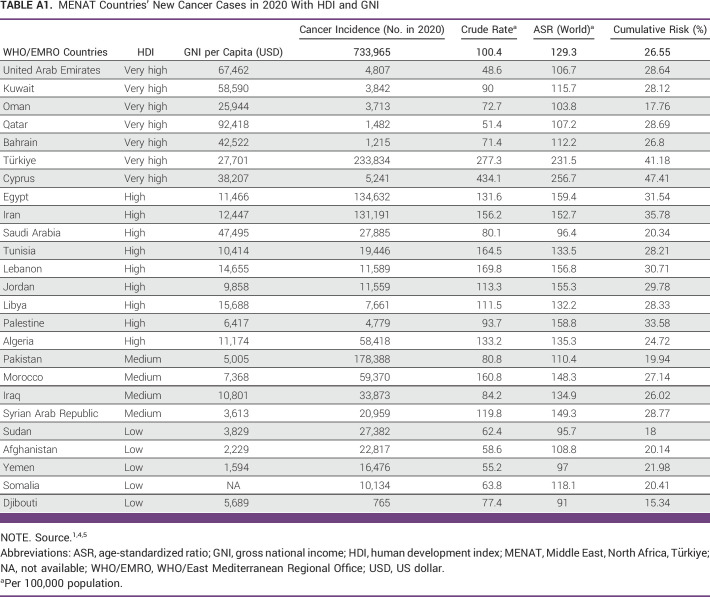
MENAT Countries' New Cancer Cases in 2020 With HDI and GNI

**TABLE A2:**
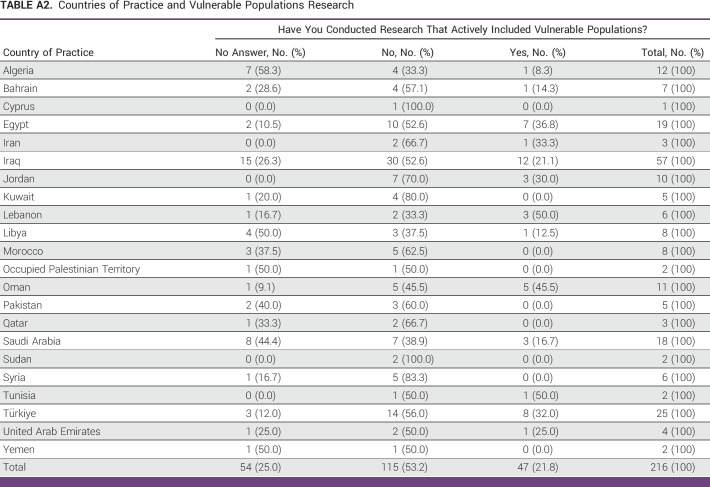
Countries of Practice and Vulnerable Populations Research

**TABLE A3:**
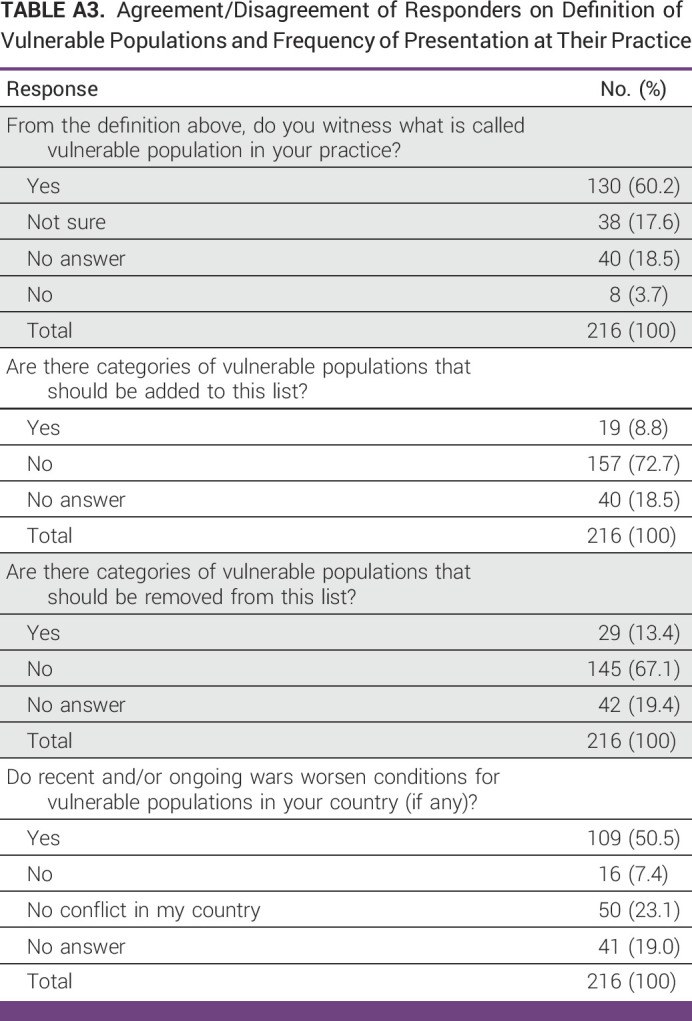
Agreement/Disagreement of Responders on Definition of Vulnerable Populations and Frequency of Presentation at Their Practice

**TABLE A4:**
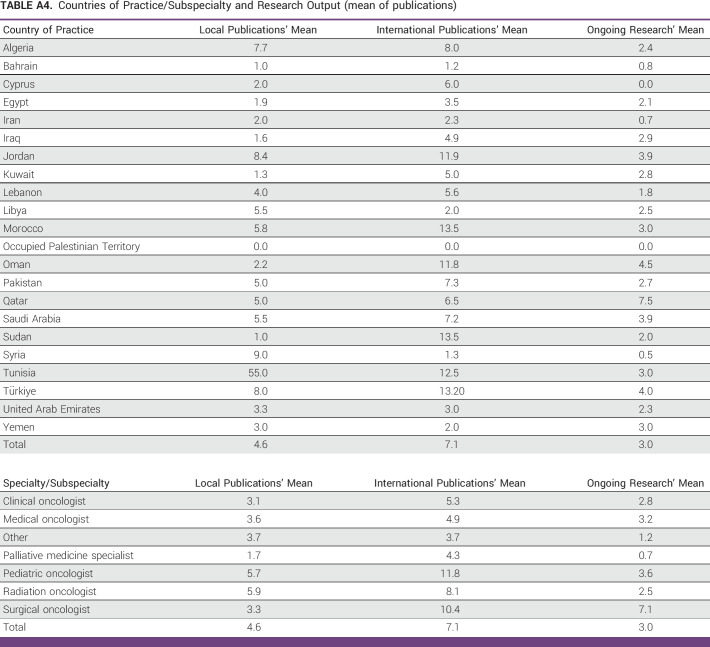
Countries of Practice/Subspecialty and Research Output (mean of publications)

## PREPRINT VERSION

Preprint version available at https://ascopubs.org/doi/abs/10.1200/JCO.2023.41.16_suppl.e13649.

## APPENDIX 1. MENA and MENAT Definition

MENA acronym refers to a region including countries extended across the Middle East and North Africa that share common historical, geopolitical, and cultural ties and challenges. MENA countries vary in human development index and gross national income per capita (Appendix Table A1). Its population represents about 11% of the total world populations (n = 858,508,884). There is no standard definition for MENA; however, different organizations use the term. According to the international monetary fund in 2003, MENA includes Arab states in the Middle East and North Africa—Algeria, Bahrain, Comoros, Djibouti, Egypt, Iraq, Jordan, Kuwait, Lebanon, Libya, Mauritania, Morocco, Oman, Palestine (West Bank and Gaza), Qatar, Saudi Arabia, Somalia, Sudan, the Syrian Arab Republic, Tunisia, the United Arab Emirates, and Yemen—plus the Islamic State of Afghanistan, the Islamic Republic of Iran, Pakistan, and Israel.^8^ Historically, countries like Türkiye and Cyprus are also sometimes included in the MENA definition, which—in our opinion—serves the wider definition and concept of the greater eastern Mediterranean region, more optimal, and inclusive, and we used MENAT accordingly.^7^

## APPENDIX 2. Vulnerable Population Categories

(1) Older Patients/Geriatrics; (2) Children/Minors/Adolescents and Young Adults; (3) Pregnant Patients; (4) Divorced Individuals/Widowed Women; (5) Migrant Populations/Internally Displaced Populations/War-affected Patients; (6) Ethnic Minorities/Geographic (Rural) Isolation; (7) Detainees/Prisoners; (8) Veterans; (9) Dementia Sufferers/Cognitively Impaired/Psychiatric Comorbidities/Psychiatric Patients/Psychosocial Vulnerabilities; (10) Addictions/Drug/Substance Abusers; (11) Organ Dysfunction Sufferers (Chronic Renal Impairment, Hepatic Impairment, Kidney Failure, Hemodialysis, Liver Failure)/Solid Organ Transplant Recipients/Patients With Auto-Immune Disease/Patients With Chronic Viral Infections (HIV, hepatitis B virus, etc); (12) Socioeconomically Deprived/Patients with Poor Performance Status/Social Isolation/Remote Isolation; (13) LGBTQ+ Population; (14) People with Limited Health Literacy/Physically Handicapped Patients; (15) Morbidly Obese Patients; (16) Terminally Ill Patients.^39^

## APPENDIX 3. Sample Statements/Answers in Response to Questions From the Survey

### Question

Are there categories of vulnerable populations that should be added to this list?

### Sample Answers

“The LGBTQ+ population tends not to disclose their sexual behavior and orientation, and that may lead to late presentation of cancer because of lack of acceptance and support”; “Maybe mentioned, but I want to emphasize on patients coming from another state/province/governorate within the country (usually poor and find problems of lodging or navigation)”; “sexually assaulted victims”; “single women living with close family members (they don't always get the care they need, and their opinions aren't taken into consideration by their families for decisions regarding their own bodies and own care)”

### Question

Are there categories of vulnerable populations that should be removed from this list?

### Sample Answers

“Divorced, widowed”; “veterans”; “divorced individuals, widowed women”; “this list means that almost all patients will be vulnerable. I think that chronic patients who are not under pressure or who are not forced to participate in specific research (ie, voluntarily) can be easily considered non-vulnerable”

### Question

In your opinion, which of these challenges is the main reason for research scarcity at your facility/country? Please comment on each option selected.

### Sample Answers

“Private hospitals don't spend money on research”; “Nobody will help you in this area”; “No budget”; “No financial support”; “No funding for important research could be offered by any governmental institute”; “Research and studies need some funding to encourage the researchers to do their best, and also the studies have some spending costs”; “No funds for radiation oncologists”; “Either no funding or insufficient funding. Too much relying on pharma”; “From government”; “Mainly cultural hesitation to enroll in trials”; “Culture still specifically female”; “The bureaucracy!”; “We don't have governing law for research”; “Difficulties in talking with patients for taking their consent”; “Difficulty in convincing patients of participation. Unable to understand the aim of the project and the importance of their participation”; “No training at all for research”; “We lack research training”; “Needs special training”; “Work time not specifically given to research”; “There is no clear specification of the time dedicated for research”; “No protected time at all”; “The pay rate is very low. It is better to spend time on active clinical practice rather than doing research to get enough money to support the family.”

## References

[1] Sung H Ferlay J Siegel RL et al Global cancer statistics 2020: GLOBOCAN estimates of incidence and mortality worldwide for 36 cancers in 185 countries CA Cancer J Clin 71 209 249 2021 33538338 10.3322/caac.21660

[2] Shah SC Kayamba V Peek RM et al Cancer control in low- and middle-income countries: Is it time to consider screening? JCO Glob Oncol 10.1200/JGO.18.00200 10.1200/JGO.18.00200 PMC6452918 30908147

[3] Wild CP Weiderpass E Stewart BW World Cancer Report: Cancer Research for Cancer Prevention https://publications.iarc.fr/Non-Series-Publications/World-Cancer-Reports/World-Cancer-Report-Cancer-Research-For-Cancer-Prevention-2020 39432694

[4] Mula-Hussain L Mahdi H Ramzi ZS et al Cancer burden among Arab world males in 2020: The need for a better approach to improve outcome JCO Glob Oncol 10.1200/GO.21.00407 10.1200/GO.21.00407 PMC9005253 35353549

[5] Mahdi H Mula-Hussain L Ramzi ZS et al Cancer burden among Arab-world females in 2020: Working toward improving outcomes JCO Glob Oncol 10.1200/GO.21.00415 10.1200/GO.21.00415 PMC8920429 35259001

[6] Pramesh CS Badwe RA Bhoo-Pathy N et al Priorities for cancer research in low- and middle-income countries: A global perspective Nat Med 28 649 657 2022 35440716 10.1038/s41591-022-01738-x PMC9108683

[7] Dumper M Stanley BE Cities of the Middle East and North Africa: A Historical Encyclopedia Santa Barbara, CA ABC-Clio 2007

[8] Davoodi MHR Abed MGT Challenges of Growth and Globalization in the Middle East and North Africa International Monetary Fund 2003

[9] Skelton M Alameddine R Saifi O et al High-cost cancer treatment across borders in conflict zones: Experience of Iraqi patients in Lebanon JCO Glob Oncol 10.1200/JGO.19.00281 10.1200/JGO.19.00281 PMC6998032 32031440

[10] Dewachi O Skelton M Nguyen V-K et al Changing therapeutic geographies of the Iraqi and Syrian wars Lancet 383 449 457 2014. 10.1016/S0140-6736(13)62299-0 24452046

[11] Machaalani M Masri JE Ayoubi LME et al Cancer Research Activity in the Arab World: A 15-Year Bibliometric Analysis 2021 In Review. https://www.researchsquare.com/article/rs-389292/v1

[12] Hamadeh RR Borgan SM Sibai AM Cancer research in the Arab world: A review of publications from seven countries between 2000-2013 Sultan Qaboos Univ Med J 17 e147 e154 2017 28690885 10.18295/squmj.2016.17.02.003 PMC5488814

[13] Albarqouni L Elessi K Abu-Rmeileh NME A comparison between health research output and burden of disease in Arab countries: Evidence from Palestine Health Res Policy Syst 16 25 2018 29544498 10.1186/s12961-018-0302-4 PMC5856204

[14] El Rassi R Meho LI Nahlawi A et al Medical research productivity in the Arab countries: 2007-2016 bibliometric analysis J Glob Health 8 020411 2018 30410737 10.7189/jogh.08.020411 PMC6220353

[15] Ismail SA McDonald A Dubois E et al Assessing the state of health research in the Eastern Mediterranean region J R Soc Med 106 224 233 2013 23761582 10.1258/jrsm.2012.120240 PMC3705424

[16] Backman G Hunt P Khosla R et al Health systems and the right to health: An assessment of 194 countries Lancet 372 2047 2085 2008 19097280 10.1016/S0140-6736(08)61781-X

[17] Silbermann M Calimag MM Eisenberg E et al Evaluating pain management practices for cancer patients among health professionals: A global survey J Palliat Med 25 1243 1248 2022 35442772 10.1089/jpm.2021.0596

[18] Castro-Ríos A Martínez-Valverde S Childhood cancer survival, 2006-2012 cohorts of Mexican Institute of Social Security beneficiaries at the central-south region of Mexico Front Oncol 12 882501 2022 35847881 10.3389/fonc.2022.882501 PMC9283836

[19] Asghar K Abu Bakar M Ashfaq S et al COVID-19 in cancer patients with diabetes in Pakistan: Clinical features and management Front Oncol 12 922579 2022 36059615 10.3389/fonc.2022.922579 PMC9434633

[20] Mrema D Ngocho JS Mremi A et al Cervical cancer in Northern Tanzania—What do women living with HIV know Front Oncol 12 957325 2023 36698389 10.3389/fonc.2022.957325 PMC9868899

[21] Tack L Schofield P Boterberg T et al Editorial: Clinical cancer research in vulnerable populations Front Oncol 13 1166714 2023 36937380 10.3389/fonc.2023.1166714 PMC10019276

[22] Bernard HR Research Methods in Anthropology: Qualitative and Quantitative Approaches Rowman Altamira 2006. https://handoutset.com/wp-content/uploads/2022/06/Russel-Research-Method-in-Anthropology-Qualitative-vs-Quantitative.pdf

[23] Campbell DT The informant in quantitative research Am J Sociol 60 339 342 1955

[24] Tongco MDC Purposive sampling as a tool for informant selection Ethnobot Res App 5 147 2007

[25] Karmel TS Jain M Comparison of purposive and random sampling schemes for estimating capital expenditure J Am Stat Assoc 82 52 57 1987

[26] Abdul-Sater Z Menassa M Achi NE et al Strengthening capacity for cancer research in conflict settings: Key informant insights from the Middle East 2020 http://ecancer.org/en/journal/article/1153-strengthening-capacity-for-cancer-research-in-conflict-settings-key-informant-insights-from-the-middle-east/abstract 10.3332/ecancer.2020.1153 PMC7864685 33574898

[27] Skelton M Al-Mash’hadani AK Abdul-Sater Z et al War and oncology: Cancer care in five Iraqi provinces impacted by the ISIL conflict Front Oncol 13 1151242 2023 37213303 10.3389/fonc.2023.1151242 PMC10196689

[28] Mula-Hussain L Cancer Care in Iraq: A Descriptive Study Riga, Latvia, LAP Lambert Academic Publishing, 2012

[29] Khoury B Rizk N Mukherji D et al LGBT populations and cancer in the Eastern Mediterranean region: Insights and challenges with a focus on Lebanon LGBT Populations and Cancer in the Global Context Cham, Switzerland Springer 2022 261 274

[30] Tolba MA Fadhil I Al-Zahrani A et al Cancer research in vulnerable populations: A call for collaboration and sustainability from MENATC countries J Clin Oncol 41 2023 suppl 16; abstr e13649 10.1200/GO.23.00201 PMC10730041 38096463

[31] Hamadeh RR Jahrami H Nazzal K Cancer research in the Arab world Al-Shamsi HO Abu-Gheida IH Iqbal F et al Cancer in the Arab World Singapore Springer Singapore 2022 395 408

[32] Abdi K Arab M Rashidian A et al Exploring barriers of the health system to rehabilitation services for people with disabilities in Iran: A qualitative study Electron Physician 7 1476 1485 2015 26767101 10.19082/1476 PMC4700893

[33] Abdul-Sater Z Kobeissi E Menassa M et al Research capacity and training needs for cancer in conflict-affected MENA countries Ann Glob Health 86 142 2020 33200073 10.5334/aogh.2809 PMC7646279

[34] Cabral BP da Graça Derengowski Fonseca M Mota FB The recent landscape of cancer research worldwide: A bibliometric and network analysis Oncotarget 9 30474 30484 2018 30093962 10.18632/oncotarget.25730 PMC6078146

[35] Benamer HTS Bakoush O Arab nations lagging behind other middle eastern countries in biomedical research: A comparative study BMC Med Res Methodol 9 26 2009 19374747 10.1186/1471-2288-9-26 PMC2674457

[36] Maziak W Geography of biomedical publications Lancet 363 490 2004 10.1016/S0140-6736(04)15500-1 14962534

[37] Sahloul E Salem R Alrez W et al Cancer care at times of crisis and war: The Syrian example JCO Glob Oncol 10.1200/JGO.2016.006189 10.1200/JGO.2016.006189 PMC5560458 28831442

[38] Abdul-Sater Z Mukherji D Adib SM et al Cancer registration in the Middle East, North Africa, and Turkey (MENAT) region: A tale of conflict, challenges, and opportunities Front Oncol 12 1050168 2022 36505790 10.3389/fonc.2022.1050168 PMC9730320

[39] Tack L Schofield P Boterberg T et al Editorial: Clinical cancer research in vulnerable populations Front Oncol 13:1166714, 2023. https://www.frontiersin.org/research-topics/32025/clinical-cancer-research-in-vulnerable-populations#main-content 36937380 10.3389/fonc.2023.1166714 PMC10019276

